# Surgicel^®^ (oxidized regenerated cellulose) granuloma mimicking local recurrent gastrointestinal stromal tumor: A case report

**DOI:** 10.3892/ol.2013.1218

**Published:** 2013-02-28

**Authors:** HAO WANG, PING CHEN

**Affiliations:** Department of Gastrointestinal Surgery, Su Bei People’s Hospital of Jiangsu Province, Yangzhou University, Yangzhou, Jiangsu 225001, P.R. China

**Keywords:** foreign body granuloma, gastrointestinal stromal tumor recurrence, Surgicel^®^ (oxidized regenerated cellulose), fludeoxyglucose (^18^F)-positron emission tomography-computed tomography

## Abstract

Unexpected clinical and/or imaging evidence of the recurrence of gastrointestinal stromal tumors soon after surgical resection may be complicated due to certain biological behavioral features of gastrointestinal stromal tumors. However, local hemostatic materials routinely used in abdominal surgery to achieve hemostasis intraoperatively may cause a foreign-body reaction, which appears to be indistinguishable from recurrent tumors in imaging studies. Thus, a second examination may be necessary to settle the true nature of the findings in such cases. If the resection and examination reveals a recurrent tumor, further proper oncological treatment is warranted, whereas if a foreign-body reaction is observed, radical or potentially harmful therapy may be withheld or cancelled. The present study retrospectively analyzes the case of an 83-year-old male patient who presented with a recurrent gastrointestinal stromal tumor four months after surgical resection, which was later identified as an intra-abdominal foreign-body granuloma caused by retained Surgicel^®^ residue. The present study aimed to demonstrate why foreign-body granuloma induced by local hemostatic materials should be incorporated into the differential diagnosis of recurrent gastrointestinal stromal tumors post-operatively, particularly soon after surgical resection has been performed.

## Introduction

Hemostatic materials [e.g., Surgicel^®^ (oxidized regenerated cellulose)] are usually spread in general surgery to assist in the control of capillary, venous and small arterial hemorrhages and oozing blood when ligation, electrical coagulation or other conventional methods of control are impractical or ineffective (1). The materials are often left in the surgical bed, as they are bio-absorbable. However, retained hemostatic materials may mimic abscesses or recurrent tumors, as inappropriate handling, marked foreign-body reactions, chronic inflammation and infections are able to cause foreign-body granuloma formation (2). The present study retrospectively analyzed the case of an 83-year-old male Chinese patient who presented with a recurrent gastrointestinal stromal tumor (GIST) four months subsequent to surgical resection. The GIST was later identified as an intra-abdominal foreign-body granuloma caused by retained Surgicel residue that mimicked a local recurrent GIST. The study was approved by the Ethics Committee of Su Bei People’s Hospital of Jiangsu Province, Yangzhou, China. The patient consented to the publication of this study.

## Case report

An 83-year-old male Chinese patient was admitted to the Department of Gastroenterology of Su Bei People’s Hospital of Jiangsu Province (Yangzhou, China) due to left upper quadrant abdominal pain lasting for 3 days. The patient complained that the pain was accompanied by nausea and vomiting post-prandially, as well as an intermittent low-grade fever for ∼4 months. The patient experienced weight loss of 3 kg, but no diarrhea, constipation, anorexia, chills or jaundice. According to the patient’s surgical history, surgical resection for GIST had been performed four months previously and right colon resection for ascending colon adenocarcinoma five years previously.

At admission, the patient’s vital signs (blood pressure, respiratory rate, temperature and heart rate) were stable. An abdominal examination revealed a solid, firm, non-tender mass in the left upper quadrant. The remainder of the examination was normal.

The only abnormal laboratory parameter was an increased neutrophil leukocyte measurement of 73.5% (normal, 42–70%). The routine laboratory parameters, including the white blood cell count, erythrocyte sedimentation rate (ESR), C-reactive protein (CRP), liver and pancreatic enzyme and tumor marker [carcinoembryonic antigen (CEA), carcinoma antigen (CA)-125, CA-199, prostate-specific antigen (PSA) and α-fetoprotein (AFP)] levels, were all within the normal limits.

**Figure 1:**
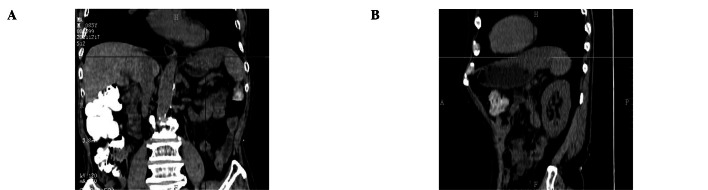
Four months subsequent to GIST resection. (A) Coronal and (B) sagittal reconstructed CT images revealing a heterogeneous mass, with a maximum diameter of 8.3 cm and a density of −24 to 35 HU, situated between the gastric fundus and spleen. The stomach wall and the upper pole of the spleen appeared to have been invaded. GIST, gastrointestinal stromal tumor; CT, computed tomography.

**Figure 2:**
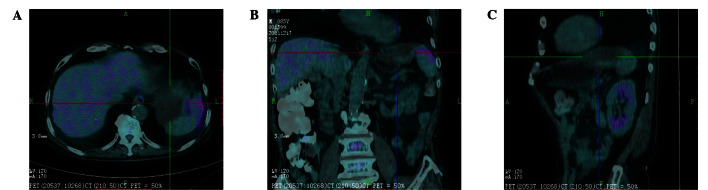
(A) Transaxial, (B) coronal and (C) sagittal PET/CT images showing an uneven, rim-shaped FDG-uptake pattern, rather than a global pattern at the tumor periphery. In the false-colour images, gray indicates no FDG-uptake, blue indicates weak FDG-uptake, purple indicates moderate FDG-uptake and red indicates marked FDG-uptake. PET/CT, positron emission tomography-computed tomography; FDG, fludeoxyglucose (^18^F).

**Figure 3:**
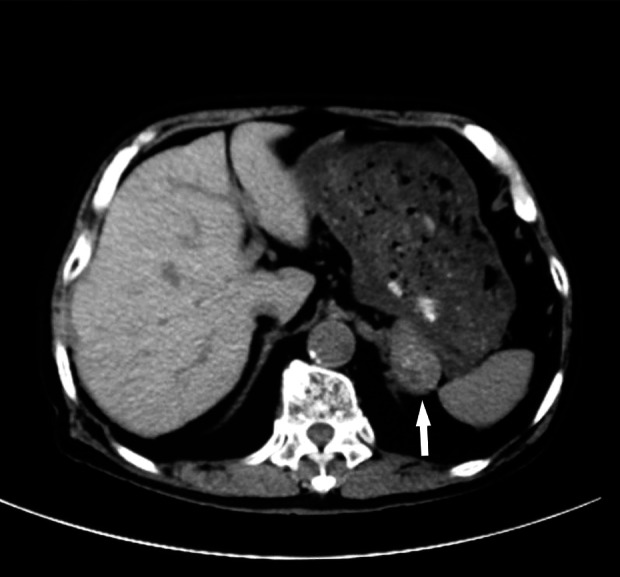
Post-contrast CT screen obtained four months previous to admission showing a homogeneous tumor (arrow) measuring ∼4×4 cm, which invaded the stomach wall. CT, computed tomography.

A computed tomography (CT) scan with intravenous contrast medium revealed a heterogeneous mass, with a maximum diameter of 8.3 cm and a density of −24 to 35 HU, located between the stomach and spleen (Fig. 1A and B). A positron emission tomography-computed tomography (PET/CT) scan also revealed a lesion with an uneven, rim-shaped pattern, rather than a global fludeoxyglucose (^18^F; FDG)-uptake pattern (Fig. 2A–C). The abdominal CT screen obtained prior to the GIST resection was reviewed and revealed a homogeneous lesion measuring ∼4×4 cm and invading the stomach wall (Fig. 3).

**Figure 4:**
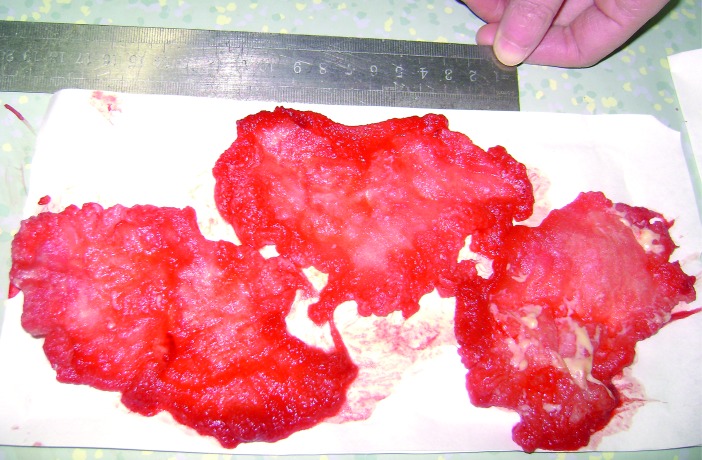
When dissected, the mass was revealed to be three pieces of retained Surgicel^®^ residue with pus.

**Figure 5:**
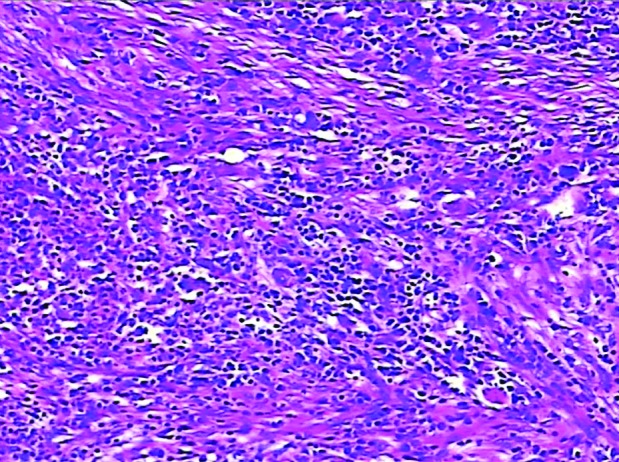
Pathological examination revealed a fibrous encapsulation containing the foreign-body giant cell reaction (H&E staining; magnification, ×100).

Taking into account the previous observations, an explorative laparotomy was performed upon clinical diagnosis of a local recurrent GIST. During the exploration, a mass wrapped in a fibrous capsule that adhered to the gastric fundus and spleen was identified, with the same diameter as measured by CT. Following en bloc removal, the mass was dissected and identified as a quantity of retained local hemostat residue and pus (Fig. 4). Microscopic examination revealed a fibrous encapsulation containing the foreign-body giant cell reaction (Fig. 5). The surgeon who performed the surgery on the patient four months previously was contacted and they acknowledged that three pieces of Surgicel (oxidized regenerated cellulose) had been implanted between the stomach and spleen intraoperatively. The patient recovered well post-operatively and was discharged on the 16th day. The patient showed no signs of recurrence at an 11-month follow-up.

## Discussion

Hemostat-associated masses, termed gauzomas, gossypibomas, textilomas or even Surgicelomas (3), are actually foreign-body reactions against retained local hemostatic residues. Unlike non-degradable gauzes, which are occasionally accidentally left in the body intraoperatively, bioabsorbable hemostatic agents (e.g. oxidized regenerated cellulose) are intentionally placed in the surgical field. Oxidized regenerated cellulose, which has been branded as Surgicel by Ethicon (Johnson and Johnson, Somerville, NJ, USA), is produced by decomposing wood pulp, then regenerating the cellulose by manufacturing continuous cellulose fibers. Since it is bioabsorbable, Surgicel is widely used to control bleeding when ligation, electrical coagulation or other conventional hemostatic methods are impractical or ineffective, and it is often left in the surgical bed. However, misdiagnoses of Surgicel granulomas mimicking recurrent tumors have resulted in secondary examinations being reported in neurosurgeries, thoracic surgeries and gynecological procedures (3–6). These cases have revealed that the exact hemostasis and dissolving mechanisms of Surgicel are often poorly understood by surgeons, leading to its inappropriate use.

Surgicel decreases the pH of its surroundings. This low pH has an antimicrobial effect against miscellaneous pathogenic organisms. However, the acidic character may also increase inflammation of the surrounding tissue and delay wound healing (2). Hill (7) reported that a small quantity of local hemostat enhanced infection. The dissolution of Surgicel depends on the quantity, site of implantation and the environmental factors, and the process may last for between two and six weeks (8). When a local hemostat is used and left intraoperatively, surgeons often assume that is absorbed promptly. However, the complicated degradation reactions of implanted local hemostats include injury, blood-material interactions, provisional matrix formation, acute or chronic inflammation, granulation tissue development, foreign-body reactions and fibrous capsule development (9). Histological evidence of oxidized cellulose fibers several years subsequent to surgery has been revealed in certain studies (5,10,11). Moreover, cases have been reported where the Surgicel used for hemorrhage control during a thoracotomy had passed through the intervertebral foramen and caused spinal cord compression (3,12). These cases indicate that only the smallest necessary quantity should be used and any excess should be removed once the hemostatic effect has been achieved to avoid post-operative foreign-body granuloma formation.

A Surgiceloma may present in either the immediate or delayed phase following surgery. The general complaints of patients may be nausea, vomiting, a palpable mass, rectal bleeding or changes in bowel habits. Surgiceloma may also present with a non-specific fever, anorexia and weight loss, which may mimic a malignant disease (13). The present case presented with classic foreign-body granuloma induced by retained Surgicel residues, which mimicked a local recurrent GIST. GISTs are the most common mesenchymal neoplasms of the gastrointestinal (GI) tract, with the most common locations being the stomach (60%), jejunum and ileum (30%), duodenum (5%) and colorectum (5%) (14). Among patients with primary GIST who underwent complete resection, 7% had an isolated local recurrence and there was a trend for tumors >10 cm to recur earlier (15). In the present case, the inappropriate use of Surgicel caused a marked foreign-body reaction and a fibrous capsule surrounding the Surgicel residues was formed. The Surgicel granuloma caused a pyloric obstruction by a mass effect, while the persistent inflammatory response resulted in a recurrent non-specific fever.

**Figure 6:**
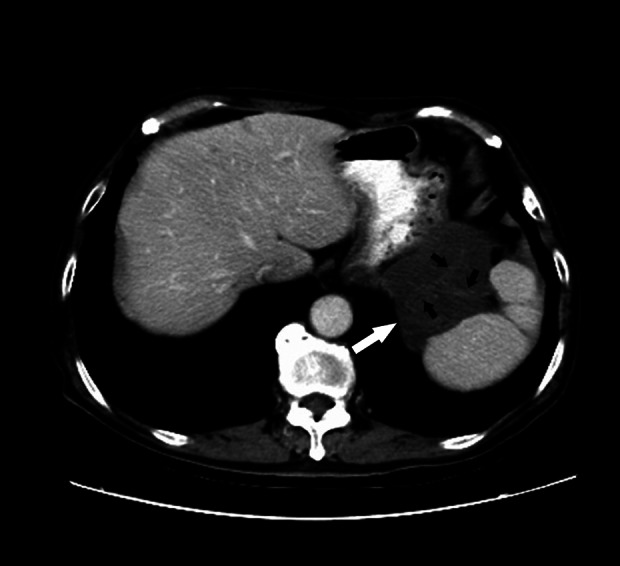
Transaxial enhanced abdominal CT scan showing an unenhanced gyrus-like mass (white arrow) which was divided into 3 parts by linear stripes (black arrow; ‘ribbon sign’).

O’Connor *et al*(16) described the CT appearances of absorbable hemostats as mixed- or low-attenuation masses containing focal central collections of gas. CT scans may reveal a faint enhancement at the tumor periphery, which further contrasts with the internal low density mass. Oto *et al*(17) described the appearance of oxidized regenerated cellulose in post-operative T2-weighted magnetic resonance (MR) images with a short relaxation time, which resulted in a low signal intensity in the early post-operative period. Yuh-Feng *et al*(18) reported that reconstructed PET/CT images may reveal an uneven FDG uptake (rim-shaped, rather than global, FDG-uptake pattern) at the mass periphery in cases of gossypiboma. Together, all these imaging observations may contribute to the identification of Surgicel granulomas and recurrent tumors. In the present case, an enhanced CT scan revealed an unenhanced gyrus-like mass (Fig. 6), with a heterogeneous density. Furthermore, the PET/CT images showed a characteristic rim-shaped uneven FDG-uptake pattern at the periphery of the ‘tumor’. These findings should arouse suspicion with regard to the diagnosis of foreign-body granuloma, since short-term local recurrence of GIST is quite rare. However, in light of the patient having had two previous surgical resections of tumors and not having received Gleevec^®^ chemotherapy, a diagnosis of recurrent GIST was made pre-operatively.

In conclusion, Surgicel granulomas are uncommon and mostly cause surgeons to provide an inaccurate diagnosis. Surgicel granuloma should be included in the differential diagnosis of new or recurrent soft-tissue masses detected in patients with a history of prior surgery.
